# Assessment of immunological and hematological parameters in *Blastocystis* species-infected chronic leukemic patients

**DOI:** 10.1186/s13099-025-00733-0

**Published:** 2025-08-11

**Authors:** Heba Elhadad, Bassam Mohamed Abdel-Fattah, Sally A. M. Saleh, Moustafa Abo El-Hoda, Hend El-Taweel

**Affiliations:** 1 Department of Parasitology, Medical Research Institute, Alexandria University, Alexandria, Egypt; 2 Medical Research Institute, Alexandria University, Alexandria, Egypt; 3 Department of Hematology, Medical Research Institute, Alexandria University, Alexandria, Egypt

**Keywords:** *Blastocystis*, Chronic lymphocytic leukemia, Coproantigen, ELISA, Immunity, CD4, IL8

## Abstract

**Aim:**

*Blastocystis* spp. is a common intestinal protozoan with controversial pathogenicity. It is frequently associated with gastrointestinal (GIT) disturbances and is particularly prevalent among immunocompromised individuals. This study aimed to assess the prevalence of *Blastocystis* spp. infection and its association with immunological and hematological parameters among chronic leukemic patients.

**Methods:**

Stool and blood samples were collected from 100 chronic leukemic patients. Microscopic examination and a coproantigen assay were performed for the detection of *Blastocystis* spp., along with assessment of anti-*Blastocystis* fecal IgA and serum IgG antibodies. CD4 T cells and the serum level of IL-8 were also measured.

**Results:**

The overall *Blastocystis* spp. infection rate was 60%, determined through combined microscopy and/or coproantigen detection. Among infected patients, anti-*Blastocystis* IgA was positive in only three patients and IgG in 18 patients, with no statistically significant association between *Blastocystis* spp. infection and detection of antibodies. Infection was significantly associated with elevated IL-8 levels and WBC count. There was no statistically significant association between the presence of gastrointestinal symptoms and the levels of anti-*Blastocystis* IgG or IgA, IL-8, or CD4 count in *Blastocystis* spp.-infected patients.

**Conclusion:**

Our study reveals a high prevalence of *Blastocystis* spp. infection among chronic leukemic patients and identifies a significant association between infection and elevated IL-8 levels.

**Supplementary Information:**

The online version contains supplementary material available at 10.1186/s13099-025-00733-0.

## Introduction

*Blastocystis* species (spp.) are anaerobic unicellular intestinal parasites with a prevalence of up to 10% in developed countries, rising to 50% in developing countries [1, 2]. This prevalence may increase remarkably to 80% in immunocompromised individuals, including those with malignancies, diabetes, and renal diseases [3]. They are highly polymorphic organisms with unclear boundaries between forms; the vacuolar form is the most common [4, 5].

*Blastocystis* spp. reside in the intestinal lumen of the ileum and caecum, adhering to the outer layer of the mucus membrane, with mucosal invasion rarely reported. It triggers both cellular and humoral immune responses, thereby increasing the inflammatory response of the intestinal mucosa [6]. Persistent and chronic inflammatory responses can be detrimental to the host, contributing to tissue damage through the release of various pro-inflammatory and anti-inflammatory cytokines, which may play a role in mutagenesis, carcinogenesis, and the development of inflammatory bowel disease [7, 8].

The pathogenicity of this protozoan remains controversial and inconclusive [9]. The infection may be asymptomatic or present with symptoms such as flatulence, diarrhea, vomiting, and abdominal pain [10]. Notably, symptomatic *Blastocystis* infections are reported more frequently in immunocompromised individuals. Immunosuppression can increase the susceptibility to infection and worsen the outcome [11, 12].

*Blastocystis* detection is commonly performed through microscopic examination of stool samples, either directly or following prior culture. Immunologic diagnostic methods based on coproantigen detection have been developed, offering a reliable alternative to microscopy and allowing for high-throughput screening with the potential for automation. Following infection, antibodies specific to *Blastocystis* antigens have been identified in both fecal and serum samples [13, 14].

Chronic lymphocytic leukemia (CLL) is the most prevalent form of leukemia in adults in developed countries, with an age-adjusted incidence of 4 to 5 cases per 100,000 of the population [15]. Infections represent a leading cause of both morbidity and mortality in leukemic patients and are often considered a leading cause of death in these patients [16]. Newly diagnosed patients with CLL often exhibit immune defects, including hypogammaglobulinemia and functional impairments in T-cells, suppressor natural killer cells, dendritic cells, neutrophils, and the complement system [17, 18]. Hypogammaglobulinemia is the main immune defect, linked to an increased risk of severe infection, with a fivefold higher risk when serum IgG levels fall below 600 mg/dL [19]. However, a clear-cut correlation between specific immunoglobulin levels and the risk of infection is not well established, and patients with normal immunoglobulin serum levels may have recurrent infections [20]. Notable alterations in the levels of interleukin-8 (IL-8), a pro-inflammatory cytokine involved in neutrophil recruitment and inflammation, and in CD4 T cell counts represent key immune defects that contribute to CLL progression [21, 22]. These abnormalities are known to increase susceptibility to a range of infections, including parasitic ones [23].

This work aims to study the prevalence of *Blastocystis* spp. infection in CLL patients and its effect on the immunological response. This will help in evaluating its pathogenicity in immunosuppressed patients.

## Materials and methods

### Study subjects

A cross-sectional study was conducted on 100 newly diagnosed CLL patients attending the inpatient clinic of the Hematology department, Medical Research Institute, Alexandria, Egypt, during the period from January 2022 to November 2023.

Exclusion criteria included patients having a history of antiparasitic, antibiotic, antidiarrheal, or laxative use within two weeks before the study.

A questionnaire recording demographic and clinical data, as well as potential risk factors, was completed for all patients.

**Fig. 1:**
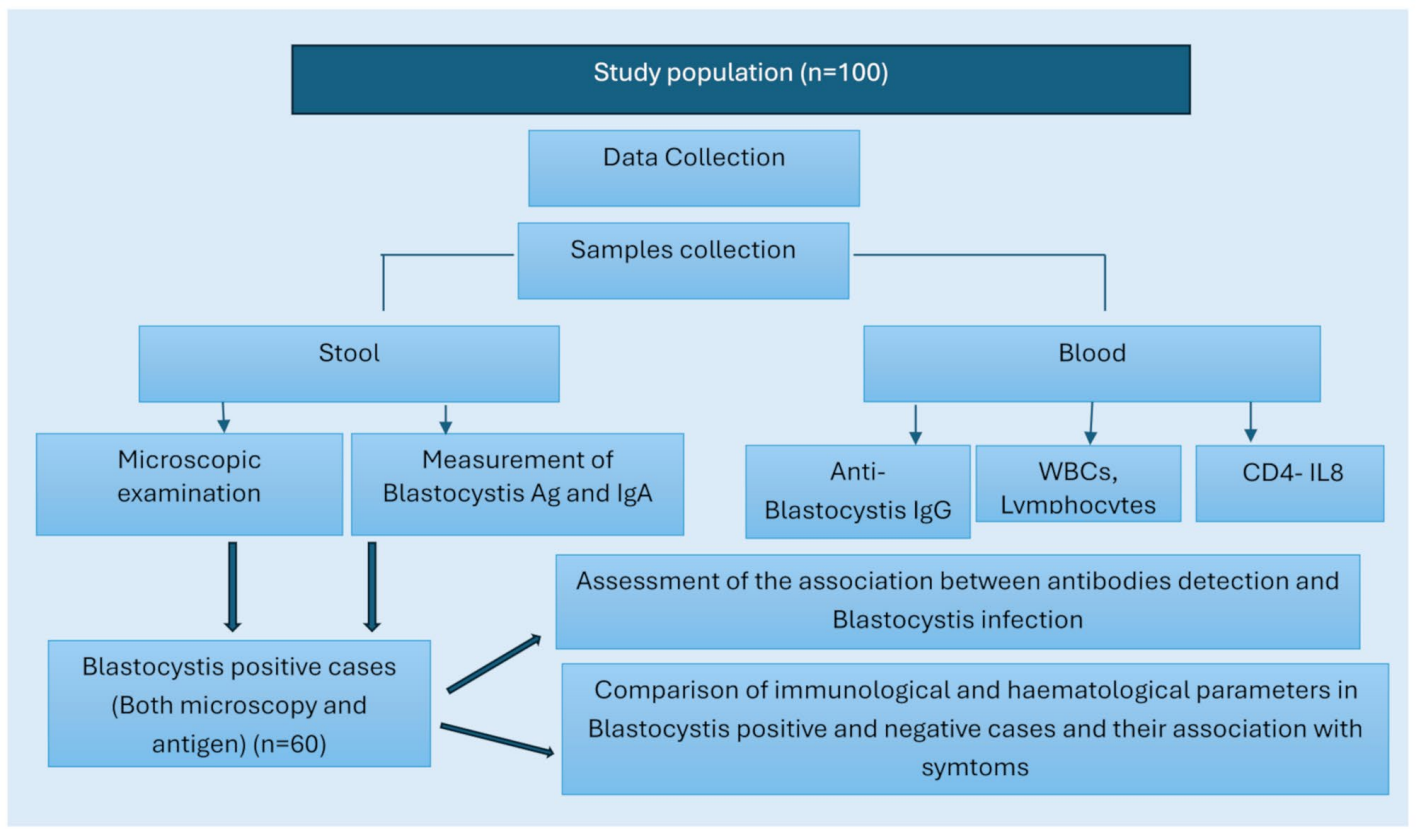
Flow chart showing the study design

### Ethical considerations

The study protocol and the template for the structured questionnaire were approved by the Research Ethics Committee, Medical Research Institute, Alexandria University, before the beginning of the study. Informed consent was approved by participants before inclusion in the study.

### Detection of *Blastocystis* infection

Fresh stool samples were collected from all study participants in sterile, labelled plastic containers. A portion of each sample was examined immediately by direct wet mount and formalin-ethyl acetate techniques for microscopic detection of *Blastocystis* spp. and other parasites [24]. Another portion was stored frozen for detection of *Blastocystis* coproantigen (Biospes, China) (Catalog No.: BZEK1785) using Enzyme-linked immunosorbent assay (ELISA) according to the manufacturer’s instructions.

### Immunological and hematological investigations

Blood samples were collected from all study participants and divided into two portions. The first portion was collected on anticoagulation tubes (EDTA) to calculate the total and differential counts of white blood cells (WBC). Peripheral blood CD4 cell count was quantified by flow cytometry [25]. The second portion was collected in a vacuum tube with gel and clot activator and left for 20 min at room temperature, and then centrifuged (3000 rpm) for 5 min. Sera were then collected and kept frozen for subsequent determination of the levels of anti-*Blastocystis* IgG (Biospes, China) (Catalog No.: BZEK1792) and interleukin-8 (IL-8) (Biospes, China) (Catalog No.: BEK1113) using ELISA techniques according to the manufacturer’s instructions.

Additionally, a third portion of each stool sample was kept frozen for determination of the level of anti-*Blastocystis* IgA (Biospes, China) (Catalog No.: BZEK1791) using ELISA according to the manufacturer’s instructions. A flow chart summarizing the study design is shown in Fig. 1.

### Statistical analysis

Data were fed to the computer and analyzed using IBM SPSS software package version 20.0 (Armonk, NY: IBM Corp, released in 2011). Qualitative data were described using numbers and percentages. Chi-square test to compare categorical variables. The Kolmogorov-Smirnov test was used to verify the normality of the distribution. Quantitative data were described using mean, standard deviation, range, median, minimum and maximum, and interquartile range (IQR). The Mann-Whitney test was used to analyse non-normally distributed quantitative variables. The kappa (κ) statistic was applied to assess agreement. The significance of the obtained results was judged at the 5% level.

## Results

### Characteristics of the study population

A total of 100 newly diagnosed CLL patients were included in the present study. Participants’ age ranged from 40 to 75 years. Regarding gender distribution, females constituted a higher proportion than males, accounting for 60% of the study population.

### *Blastocystis* infection in CLL patients

**Fig. 2:**
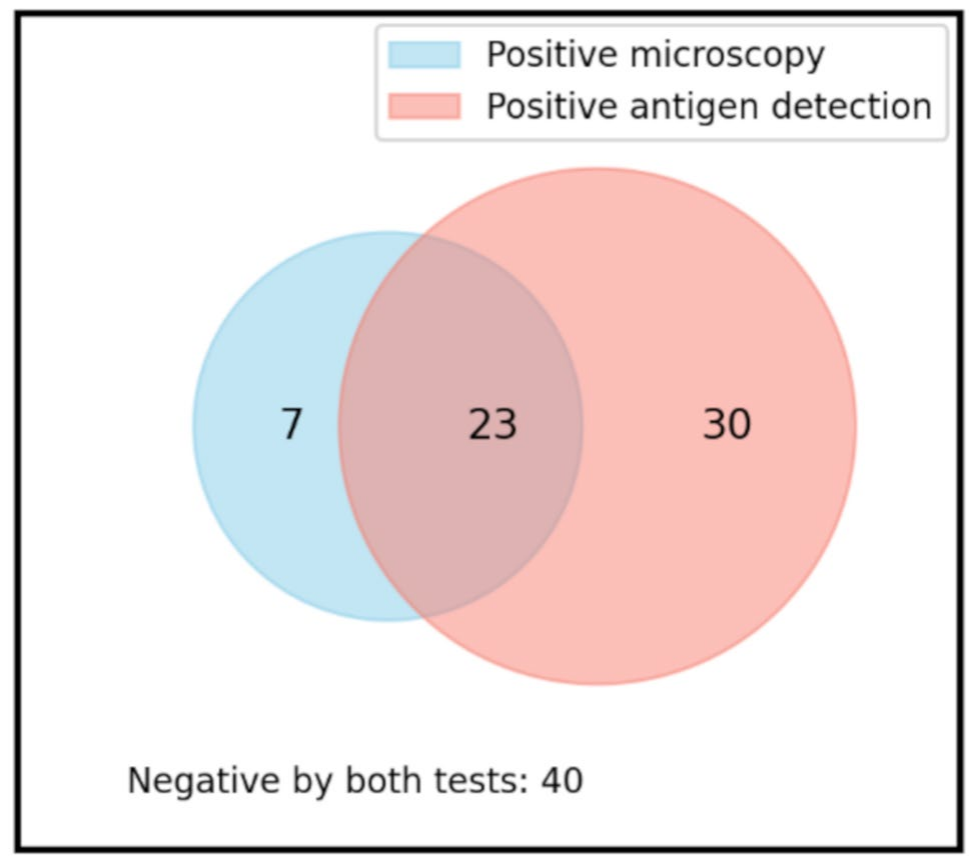
*Blastocystic* infection among chronic leukemic patients (*n* = 100) using microscopic examination and antigen detection methods. There was a fair agreement between the two tests

Among the 100 examined participants, *Blastocystis* spp. was detected in 30% of cases by microscopic examination and in 53% of cases by coproantigen detection, with 23 concordantly positive samples. The overall *Blastocystis* spp. infection rate was 60% [95% confidence interval (CI) = 50.4%-, 68.9%] using combined antigen and microscopy. Discordant results were observed in 37 samples, testing positive by one method and negative by the other. Kappa analysis indicated a fair agreement between the two methods (k = 0.277) (Fig. 2).

### Immunological and hematological parameters in *Blastocystis* infection

**Fig. 3:**
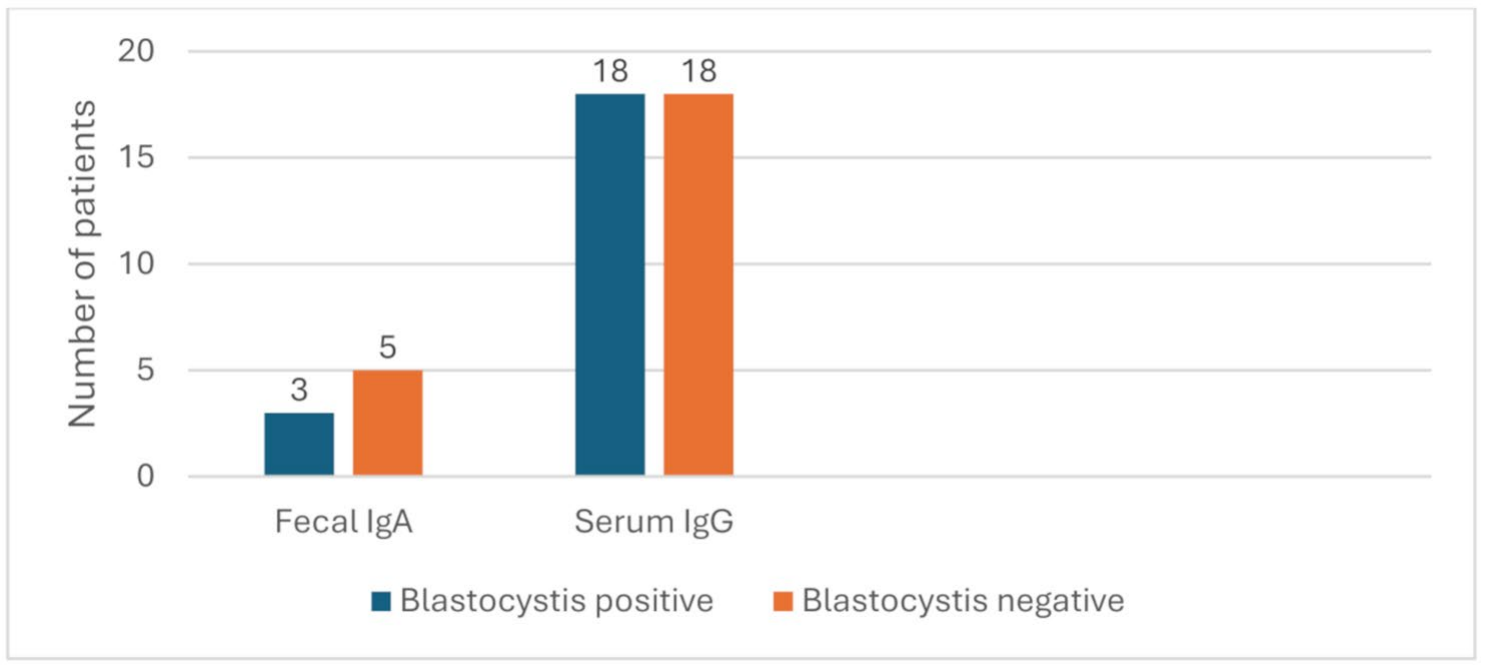
Relation between *Blastocystis* spp. infection and the detection of specific fecal IgA and serum IgG among chronic leukemic patients

Fecal IgA to *Blastocystis* was positive in only 8% of patients, while serum IgG to *Blastocystis* was seropositive in 36 cases (Fig. 3).

Among 60 patients with *Blastocystis* infection, anti-*Blastocystis* IgA level was positive in only three patients, and IgG in 18 patients. There was no statistically significant association between *Blastocystis* spp. infection and a positive antibody test.

Serum IL-8 levels were significantly higher (*p* = 0.034) in *Blastocystis*-infected patients (median: 260; IQR: 203.4-471.3; 95% CI: 22.1-1269.2 pg/mL) compared to *Blastocystis*-negative ones (median: 113; IQR: 60.2-334.5; 95% CI: 7.1-1142.2 pg/mL). A statistically significant difference was observed in WBC count between *Blastocystis*-positive and -negative patients. *Blastocystis-*infected patients presented with a median WBC count of 7000 cells/µL (IQR:5400–11300; 95%), whereas *Blastocystis*-negative patients had a median count of 5800 cells/µL (IQR: 4900–7100) (*p* = 0.025). There was no relation between *Blastocystis* spp. infection and lymphocyte or CD4 counts (Table 1 and supplementary file).

Gastrointestinal (GIT) symptoms, including diarrhea, nausea, and vomiting, were reported by 15 out of 60 patients (25%) with confirmed *Blastocystis* infection and by 14 out of 40 non-infected patients (35%), with a non-statistically significant difference between both groups. Table 2 shows that, among *Blastocystis* spp.-infected patients, there was no statistically significant relation between the presence of GIT symptoms and anti-*Blastocystis* IgA or IgG levels, IL-8, or CD4 counts.

Comparisons made using the Chi-square test revealed a non-significant difference between infected and non-infected patients regarding both parameters. (*P* > 0.05). Faecal anti-*Blastocystis* IgA in the study population ranged from 0.16 to 95.39 pg/mL, positive level ≥ 3.75 pg/mL. Serum anti-*Blastocystis* IgG levels ranged from 0.13 to 716.2 pg/mL, positive level ≥ 7.5 pg/mL.

**Table 1:** Immunological parameters in *Blastocystis* spp.-infected and non-infected chronic leukemic patients (*n* = 100)

	Blastocystis spp.		U	*p*
	Negative (*n* = 40)	Positive (*n* = 60)		
**IL-8 (pg/mL)**				
Mean ± SD.	296.4 ± 357.3	369.4 ± 322.4	899.0	0.034^*^
Median (IQR) (Min. – Max.)	113 (60.2- 334.5) (4.14–1281.3)	260 (203.4-471.3) (2.71–1459.9)		
**WBCs (x10** ^**3**^ **cells/µL)**				
Mean ± SD.	25.20 ± 105.6	21.18 ± 86.50	882.5	0.025^*^
Median (IQR) (Min. – Max.)	5.80 (4.9–7.1) (1.50–669.0)	7.0 (5.4–11.3) (0.34–675.0)		
**Total lymphocyte (x10** ^**3**^ **cells/µL)**				
Mean ± SD	3.20 ± 5.10	11.27 ± 65.53	1063.5	0.336
Median (IQR) (Min. – Max.)	1.85 (1.3–2.6) (0.28–30.0)	2.30 (1.5–3.1) (0.6 – 510.0)		
**CD4 (cells/µL)**				
Mean ± SD.	601.07 ± 341.58	630.22 ± 366.84	1157.0	0.762
Median (IQR) (Min.– Max.)	550 (388.8- 776.3) (160–2037)	575.0 (380–895) (85.0–1628)		

U: Mann-Whitney test

*: Statistically significant at *p* ≤ 0.05

**Table 2:** Parameters associated with Gastrointestinal symptoms in *Blastocystis* spp.-infected leukemic patients

	*Blastocystis* spp. (*n* = 60)		U	*P*
	Symptomatic (*n* = 15)	Asymptomatic (*n* = 45)		
**IgG (pg/mL)**				
Mean ± SD.	7.79 ± 8.65	36.85 ± 148.31	285.0	0.368
Median (IQR) (Min.– Max.)	1.2 (0.3–18.3) (0.17–19.54)	1.00 (0.24–11.7) (0.13–716.17)		
**IgA (pg/mL)**				
Mean ± SD.	7.91 ± 23.57	2.23 ± 5.72	239.0	0.052
Median (IQR) (Min.– Max.)	1.00 (1.0–3.7) (0.68–93.01)	1.0 (1.0–1.0) (0.0.17–28.39)		
**CD4 (cell/µL)**				
Mean ± SD.	631.33 ± 459.96	629.84 ± 336.35	308.5	0.620
Median (IQR) (Min.– Max.)	380 (320.0–1075.0) (190.0–1600.0)	580.0 (390.0–870.0) (85.0–1628.0)		
**IL-8 (pg/mL)**				
Mean ± SD.	449.95 ± 440.83	342.59 ± 273.15	312.0	0.663
Median (IQR) (Min.– Max.)	252.7 (209.9–602.7) (68.4–1459.9)	259.9 (201.3–468.4) (2.71–1281.3)		

U: Mann-Whitney test

## Discussion

*Blastocystis* spp. are ubiquitous parasites with a worldwide distribution. The prevalence of *Blastocystis* spp. infection varies from country to country and among different communities within the same country [1, 26]. However, the question of whether it is a commensal or pathogenic parasite under certain conditions remains unresolved [6]. Many studies about the impact of *Blastocystis* spp. in immunocompromised individuals are available [10, 27]. Microscopic examination revealed that 30% of patients included in the present study had *Blastocystis* spp. infection. A similar finding was reported in Iran by Salehi Kahyesh, Alghasi [28]. Labania, Zoughbor [29], through a case-control study, reported that cancer patients have a higher risk of *Blastocystis* infection compared to cancer-free individuals. Sulżyc-Bielicka, Kołodziejczyk [30] reported that the prevalence of *Blastocystis* spp. was five times higher in colorectal cancer patients (12.5%) than in the control group (2.42%), with a significant difference. On the other hand, Essa et al. reported contradictory results and attributed their findings to the reduced exposure of leukemic children to pathogens due to lower physical activity and increased attention to food and hygiene [31].

In our study, the coproantigen detection assay identified more positive cases compared to the routinely used microscopic methods. However, a few cases were missed by the antigen assay, and there was fair agreement between these two techniques. The sensitivity of microscopic methods may be low if parasites are present in low numbers. Formalin ethyl acetate was reported to destroy some organisms during processing, potentially reducing diagnostic efficiency [32]. Additionally, irregular parasite shedding may result in a false-negative diagnosis if only one stool sample is examined. Previous studies indicated a low sensitivity of the wet mount method, ranging from 18 to 35%. Fecal antigen detection assays have shown higher sensitivities of 82–88% with no cross-reactivity with other intestinal pathogens [33, 34]. Nevertheless, false negative results may occur if antigen levels drop below the assay’s detection limit [2].

Overall, *Blastocystis* spp. was found to be highly prevalent among CLL patients, which could be attributed to the profound immune dysfunction associated with the disease [35]. A growing body of evidence indicates that *Blastocystis* colonization involves a complex interplay with the intestinal epithelium and the underlying immune system [6, 36].

Exposure to the parasite antigens elicits an antibody response [2]. IgA is the most abundant mucosal antibody that has a fundamental function in conserving homeostasis with the microbiome by binding and neutralizing invading pathogens near the mucus layer [37]. IgA secretion in the intestinal lumen is caused by parasitic infections to limit the parasite burden and enhance immune protection [38]. In the present study, only three out of 60 patients with confirmed *Blastocystis* infection tested positive for fecal IgA. Low secretory IgA despite active infection in the study population can be attributed to several factors. Decreased immunoglobulin production occurs in CLL, and it is often related to disease stage and duration (Ravandi and O’Brien 2006). This deficiency is likely due to direct interactions between malignant B cells and other immune cells, as well as the release of cytokines with inhibitory effects on immunoglobulin synthesis [39]. Additionally, *Blastocystis* spp. are known to secrete serine proteases capable of degrading secretory IgA [40], potentially contributing to lower fecal IgA levels. A previous study reported that individuals colonized with *Blastocystis* exhibited lower levels of fecal IgA compared to those who were not colonized [41]. Degradation of secretory IgA may lead to gut dysbiosis, which in turn increases susceptibility to intestinal protozoa, including *Blastocystis* spp [42, 43]. The antibody response is also influenced by the duration of exposure and antigenic variation among different parasite subtypes [2]. Notably, the presence of a positive IgG antibody response in a substantial number of patients included in the present study, despite negative stool tests, suggests resolved previous exposure.

IL-8 plays a key role in inflammatory processes by attracting polymorphonuclear leukocytes to sites of inflammation and activating monocytes [44, 45]. The present study revealed a significant elevation of IL-8 serum levels in *Blastocystis* spp.-infected patients. Supporting this, a previous study reported that culturing of in vitro cell lines in the presence of *Blastocystis* promotes the production of IL-8 along with granulocyte-macrophage colony-stimulating factor [46]. Another study demonstrated that cysteine proteases secreted by the central vacuoles of *Blastocystis* stimulate NF-κB-mediated IL-8 gene expression in epithelial cells of the colon [47]. Elevated IL-8 levels in CLL patients indicate that *Blastocystis* spp. can trigger a proinflammatory response, contributing to GIT irritation and related symptoms. These elevated levels are consistent with findings in other pathogenic protozoan infections such as amoebiasis and cryptosporidiosis, supporting the potential pathogenic role for *Blastocystis* spp [48–50].

The median total WBC count was higher in *Blastocystis*-infected patients compared to non-infected ones, despite a higher mean WBC count in the non-infected group. This discrepancy is attributed to the skewed distribution of WBC counts, making the Mann-Whitney U test a more appropriate statistical approach. The test showed a significant elevation in WBC count among infected patients, possibly indicating a low-grade inflammatory response or subclinical co-infections. However, no significant difference was observed in lymphocyte counts between the two groups.

Although a low CD4 count is known to predispose individuals to many enteric infections, no statistically significant association with *Blastocystis* infection was observed in the present study. Similarly, Yulfi et al. (2021) reported no significant relationship between *Blastocystis* spp. infection and CD4 cell count in HIV patients presenting with diarrhea [42]. In another study, the average CD4 count in *Blastocystis*-infected HIV patients was 453 cells/µL, which is slightly below the lower limit of the reference range for healthy individuals [51].

The pathogenic potential of *Blastocystis* is related to several factors, such as the host’s immune status, the interaction of *Blastocystis* with the intestinal microbiota, and the infecting subtype. Studies carried out in different population groups suggest that *Blastocystis* can colonize the human intestinal tract and persist for prolonged periods without causing disease [11, 52]. In the present study, there was no statistically significant association between the presence of GIT symptoms and *Blastocystis* infection. Among symptomatic patients, symptoms were not related to the studied immunologic parameters. Many authors have suggested that symptomatic individuals show no correlation with *Blastocystis* spp. positivity [53, 54]. In contrast, some studies have confirmed a significant link between *Blastocystis* spp. infection and the occurrence of GIT symptoms [55, 56]. Mahmoud and Saleh reported that specific secretory IgA was not detected in asymptomatically infected individuals, whereas it was present in all cases of symptomatic *Blastocystis* spp. infection [57]. This discrepancy remains controversial, but it may be attributed to the wide variety of isolated subtypes and host defense factors such as age and immune status.

One of the limitations of this study is its cross-sectional design, which restricts the ability to establish a causal relationship between *Blastocystis* spp. infection and the observed immunological or hematological alterations. While associations were identified, it remains unclear whether *Blastocystis* infection contributes to these immune changes or whether the altered immune status in chronic leukemic patients predisposes them to infection. Longitudinal studies are needed to clarify the direction and nature of this relationship.

Conclusions: Our findings demonstrate that *Blastocystis* spp. infection is frequent among leukemic patients, but secretory IgA was detected in only a few infected cases. Serum IL-8 levels and WBC counts were significantly elevated in infected patients. Nevertheless, infection was not significantly associated with GIT symptoms or CD4 count. These results highlight the potential clinical relevance of infection by *Blastocystis* spp. in immunosuppressed patients and underscore the need for further research into its pathogenicity and diagnostic approaches.

## Supplementary Information

Below is the link to the electronic supplementary material.

Supplementary Material 1

## Data Availability

The data that support the findings of this study are available from the authors upon reasonable request.
