# Supplementary material for: Assessment of immunological and hematological parameters in Blastocystis species-infected chronic leukemic patients
Source: Gut Pathog. 2025 Aug 11;17:58. doi: 10.1186/s13099-025-00733-0 (PMC12337449; doi:10.1186/s13099-025-00733-0)

| 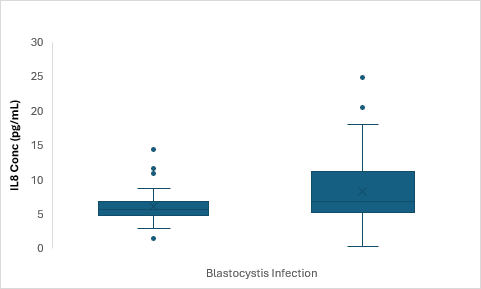  Positive  Negative | 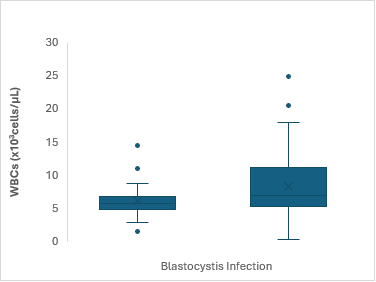  Positive  Negative |
| --- | --- |
| 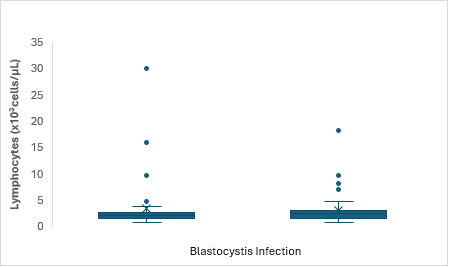  Positive  Negative | 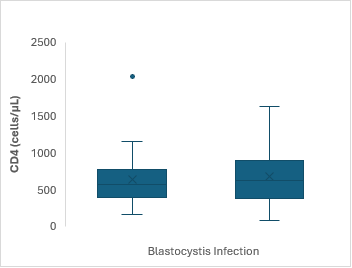  Positive  Negative |

**Immunological parameters in *Blastocystis* spp.-infected and non-infected chronic leukemic patients (n = 100).** A significant difference was detected in IL-8 concentration (p=0.034) and WBC count (p=0.025) using Mann-Whitney test

| 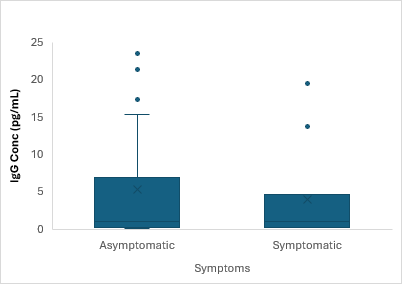 | 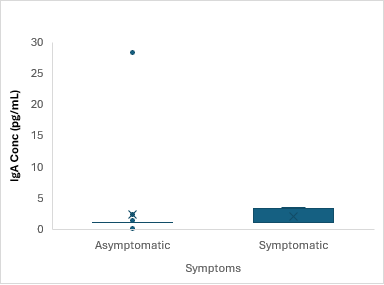 |
| --- | --- |
| 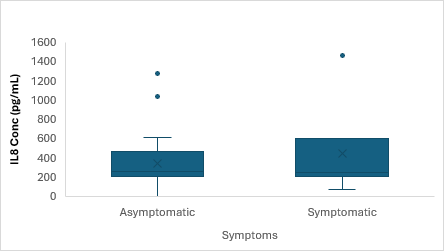 | 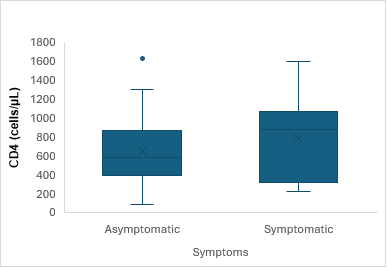 |

**Parameters associated with gastrointestinal symptoms in *Blastocystis* spp.-infected chronic leukemic patients (n=100)**


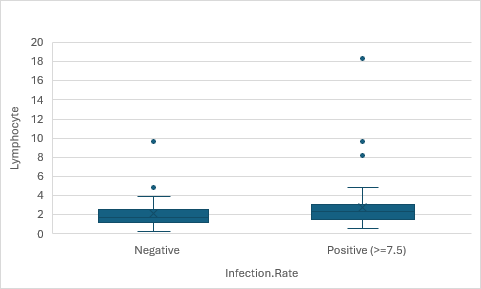

Supplement: Supplementary file 1 — Supplementary Material 1 [file 13099_2025_733_MOESM1_ESM.docx]
